# Consumption of Ultra-Processed Foods in the Brazilian Amazon during COVID-19

**DOI:** 10.3390/nu16132117

**Published:** 2024-07-02

**Authors:** Elyecleyde Katiane da Silva Oliveira, Tamires dos Santos Vieira, Orivaldo Florêncio de Souza, Priscilla Rayanne e Silva Noll, Italla Maria Pinheiro Bezerra, Matheus Paiva Emidio Cavalcanti, Luiz Carlos de Abreu, Andres Ricardo Perez Riera

**Affiliations:** 1 Post-Graduation Program in Health Sciences, University Center FMABC, Santo André 09060-650, SP, Brazil; 2 Server Health Surveillance Coordination, Federal University of Acre, Rio Branco 69920-900, AC, Brazil; 3 Post-Graduation Program in Health and Nutrition, Federal University of Espírito Santo, Vitória 69920-900, ES, Brazil; tamiresvieiraalim@gmail.com; 4 Center for Health Sciences and Sports, Federal University of Acre, Rio Branco 69920-900, AC, Brazil; orivaldo.souza@ufac.br; 5 Department of Education, Instituto Federal Goiano-Campus Ceres, Ceres 76300-000, GO, Brazil; 6 Graduate Program in Public Policies and Local Development, Superior School of Sciences of the Santa Casa de Misericórdia, Vitória 29027-502, ES, Brazil; 7 Post-Graduate Program in Medical Sciences, Faculty of Medicine, University of São Paulo, São Paulo 01246-904, SP, Brazil; mpaivaemi@usp.br; 8 Department of Integrated Health Education, Federal University of Espírito Santo, Vitória 29043-900, ES, Brazil; 9 COVID-19 Observatory Brazil and Ireland, University of Limerick, V94 T9PX Limerick, Ireland; 10 Laboratory of Studies Design and Scientific Writing, Postgraduate Division, University Center FMABC, Santo André 09060-650, SP, Brazil; riera@uol.com.br

**Keywords:** COVID-19, pandemics, ultra-processed food, food insecurity

## Abstract

Background: A COVID-19 pandemic erupted, causing a global viral pneumonia outbreak, marking the most significant public health crisis of the 21st century. These changes profoundly impacted population health and well-being, leading to shifts in dietary habits. This study aimed to evaluate the consumption of ultra-processed foods in the Brazilian Amazon before, during, and after the COVID-19 pandemic. Methods: This is a secondary data analysis study derived from the Surveillance System of Risk and Protective Factors for Chronic Diseases by Telephone Survey (Vigitel, 2019–2021) of the Brazilian Ministry of Health. All statistical analyses were performed using the Stata 17 statistical program in the survey module (svy). Results: We found an increased frequency in the subgroups of consumption of ultra-processed foods in the capital of the Brazilian Amazon region between the years 2019 and 2021. In the cities of Boa Vista and Macapá, there was a significant increase in the consumption of snacks, salty snacks, cookies, and meat products. Boa Vista and Macapá showed an increase in the percentage difference in the consumption ≥5 of ultra-processed subgroups, being 30.4% (*p* = 0.014) and 53.7% (*p* = 0.014), respectively. Conclusions: The study indicated an increase in the consumption of ultra-processed foods in the Brazilian Amazon region during and after social distancing.

## 1. Introduction

The onset of the COVID-19 pandemic precipitated a severe acute respiratory syndrome, which disseminated globally and emerged as the most significant public health crisis of the 21st century.

The World Health Organization (WHO) recommended social distancing measures to reduce the spread of the virus [1]. And this resulted in the closure of schools, universities, non-essential businesses, gyms, leisure activities, and bars, among others [2].

In Brazil, these measures were implemented in March 2020 [1], and in addition to all the problems involving this health crisis in dietary patterns [3], a reduction in physical activity time [4,5], an increase in screen time exposure [6], and changes in purchasing and consumption patterns of unhealthy foods [7] were observed. And beyond that, increased stress [8], the economic crisis [9], and unemployment [10,11]. Food insecurity also marked this period [12].

Such changes significantly affected the well-being and health of the population during the pandemic season. Specifically, in recent years, changes in food consumption patterns have been an alarming concern around the world.

In this study [13], a clear trend was observed in the increased consumption of sweets (chocolates, candies, cookies, pastries, cakes, desserts, and confectionery), snacks (packaged fatty or salty snacks), and bakery products (packaged bread, pizzas, and sandwiches), all classified as UPF, driven by the SARS-CoV-2 lockdown.

In general, eating habits, especially the consumption of ultra-processed foods, have been frequently observed, consequently resulting in an increase in metabolic and cardiovascular diseases [14] and all-cause mortality [15,16]. 

The new classification proposed by the research group led by Carlos Monteiro categorizes foods based on the level of processing and the addition of industrial ingredients and other cosmetic additives [17]. It is important to note that this classification is included in the food guide for the Brazilian population and has been recognized internationally [18].

Particularly in the Brazilian Amazon, it presents significant challenges in terms of socioeconomic development, exacerbating its vulnerability in terms of food security and consumption levels compared to other parts of the country, such as the Southeast [19]. 

Investigating consumer behavior in different regions, especially in the North, is crucial to supporting proposals for effective changes. Analysis of these changes can provide valuable information for the development of public health strategies to promote healthier food choices and mitigate adverse impacts on the health of the population.

In this context, this study aimed to evaluate the consumption of ultra-processed foods (UPF) in the Brazilian Amazon before, during, and after the COVID-19 pandemic.

## 2. Materials and Methods

### 2.1. Data Source

This is a cross-sectional study of adults in which secondary data from 2019, 2020, and 2021 were analyzed, obtained from the Brazilian Ministry of Health’s Surveillance System for Risk and Protective Factors for Chronic Diseases by Telephone Survey (Vigitel, 2019, 2020, 2021).

Data collection was carried out via telephone call with Brazilian adults from all capitals and the Federal District of Brazil. The frequency of this study is annual and has occurred since 2006, with the exception of 2022. Currently, data from 2019 (before social distancing in Brazil), 2020 (during social distancing), and 2021 (after social distancing) were analyzed.

### 2.2. Sample Plan and Eligibility Criteria

The sampling procedures are presented in Vigitel [20]. In summary, in each of the capitals of the 26 Brazilian states and the Federal District, a minimum sample size of between 1500 and 2000 adult individuals (≥18 years of age) was selected using probabilistic sampling. All the selected participants lived in households with at least one landline telephone. In 2020 and 2021, the minimum sample size was 1000 individuals in each city.

The inclusion criteria were adults aged 18 or over and living in a household with at least one landline telephone. The exclusion criteria were telephone lines located in companies and telephone lines that did not respond to six attempted calls at any time and day of the week.

**Figure 1:**
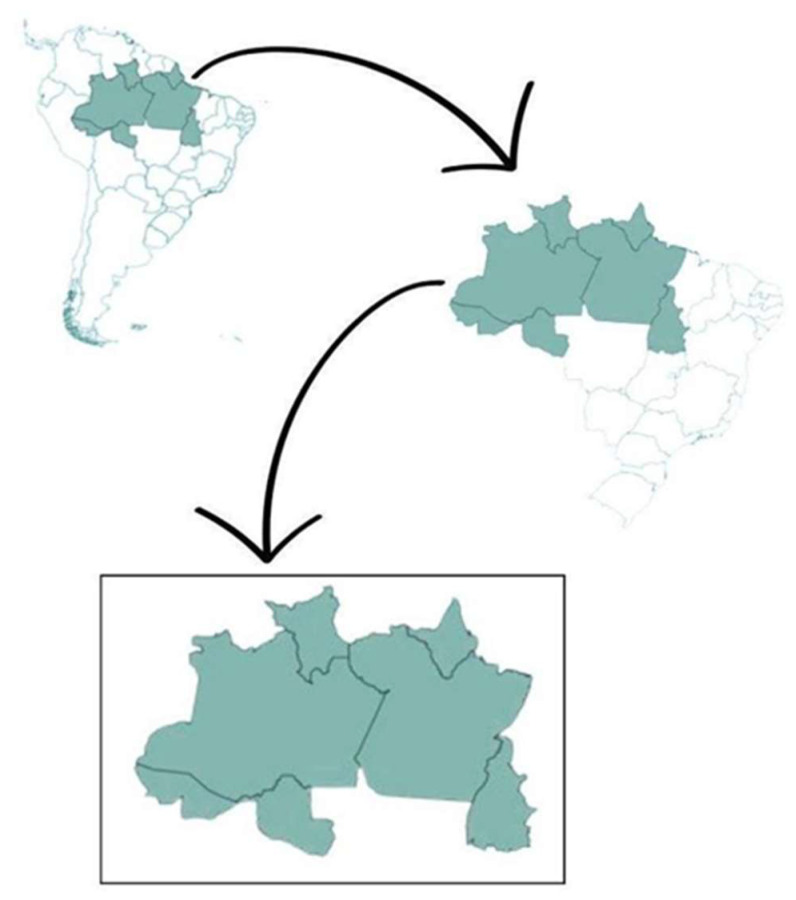
Geographic location of the Brazilian Amazon.

In this study, subsamples of adults living in capitals located in the Brazilian Amazon region were used, and the same eligibility criteria as the general sample were followed. States that are part of this region are Belém, Boa Vista, Macapá, Manaus, Palmas, Porto Velho, and Rio Branco (Figure 1).

### 2.3. Data Collection

The 2019, 2020, and 2021 databases were transferred from the Ministry of Health website (https://svs.aids.gov.br/daent/acesso-a-informacao/inqueritos-de-saude/vigitel/ (accessed on 30 September 2022) in the xls format of the Excel spreadsheet. After data collection, they were tabulated in Excel in three databases that were grouped.

The Vigitel system questionnaire is made up of questions that address a series of lifestyle issues, such as smoking, alcohol consumption, diet, and physical activity, among other risk factors for chronic diseases. In this study, we only used questions related to sociodemographic characteristics (e.g., gender, age, and education) and the participants’ self-reported food consumption.

Food consumption was assessed by asking respondents the following question: “Now I am going to list some foods, and I would like you to tell me if you ate any of them yesterday (from when you woke up to when you went to sleep)” [20]. This was performed using the 24 h recall method, with a single assessment conducted among the study population during the interview. 

The foods analyzed in this study were ultra-processed foods, whose ingredient formulations result from industrial procedures and have better palatability [21] and durability [22] classified according to the Nova classification [23].

The score for ultra-processed foods was calculated by summing the subgroups, with a range from 0 to 13. Subsequently, the score was categorized into two groups: consumption diary of less than 5 subgroups and consumption equal to or greater than 5 subgroups. To estimate the prevalence of high consumption of ultra-processed foods, the criteria of consumption of >5 different foods from the subgroup of this category on the previous day were considered [24].

**Figure 2:**
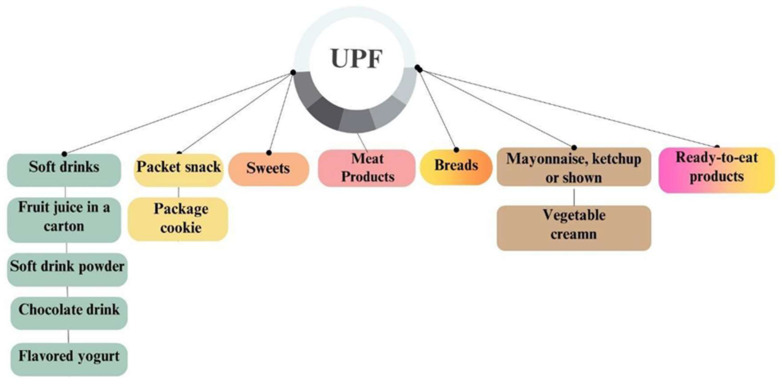
Ultra-processed foods group from the Vigitel questionnaire.

In this study, concerning the variable of ultra-processed food consumption, thirteen subgroups were delineated (Supplementary Table S1), in which aggregation occurred in some foods represented in Figure 2.

### 2.4. Data Analysis

All statistical analyses were carried out using the Stata 17 program in the survey module (svy). The relative frequencies and respective confidence intervals in 95% of the sociodemographic characteristics for the years 2019, 2020, and 2021 were calculated by the tabulate one-way command.

The prevalence and respective confidence intervals in 95% of consumption of selected subgroups from ultra-processed foods, and for each city studied, the prevalence and respective confidence intervals in 95% of consumption of ≥5 or more subgroups, in the years 2019, 2020, and 2021, were calculated by the proportion command. Subsequently, the “nlcom” command uses the Wald Test to perform hypothesis testing. Percentage differences in prevalence were calculated by the “nlcom” post-estimate command between the years 2019, 2020, and 2021, using the formula: ((final year − initial year)/initial year) × 100. The *p*-values ≤ 0.05 were considered statistically significant. 

### 2.5. Ethical Aspects of Research

This research is exempt from approval by the Research Ethics Committee because it is publicly accessible secondary data and does not identify the states or any variable that identifies the participants. 

## 3. Results

The total sample of this study in the years 2019 (n = 11,210), 2020 (n = 7030), and 2021 (n = 7022) was composed of 25,262 participants. The interviewees were female (52.25%), aged between 25 and 34 (28.27%), and lived in the capitals of the northern region. The distribution of the population according to sociodemographic characteristics in the years 2019, 2020, and 2021 can be found in the Supporting Material (Supplementary Table S2).

Table 1 presents the prevalence of consumption of ultra-processed foods and their respective confidence intervals in the seven capitals of the Brazilian Amazon region.

In 2019, the top five most prevalent foods in terms of consumption were: vegetable cream, breads, soft drinks, packet snacks or salty crackers, and ready-to-eat products. On the other hand, between 2019 and 2020, there was an increase in the consumption of all items except soft drinks, chocolate drinks, and sweets. For the years 2019 to 2021, all subgroups increased, with the exception of boxes of juice (fruit juice in a carton), soft drink powder, chocolate drink, and flavored yogurt.

Table 2 displays the percentage difference in the consumption of ultra-processed food subgroups.

When comparing the consumption of “soft drinks” in 2020 and 2019, it is possible to observe a reduction of 10.8% (*p* = 0.014). Regarding the difference in the consumption of packet snacks or salty crackers, there was a significant increase of 15.7% (*p* = 0.007) in 2021 and 2019. Sweet cookies had two increases, in 2020 and 2019 of 14.7% (*p* = 0.027) and in 2021 and 2019 of 16.2% (*p* = 0.019). Still, in relation to meat products, an increase of 16,0% (*p* = 0.010) was demonstrated between the years 2020–2019 and 14.0% (*p* = 0.021) in the years 2019 and 2021.

**Figure 3:**
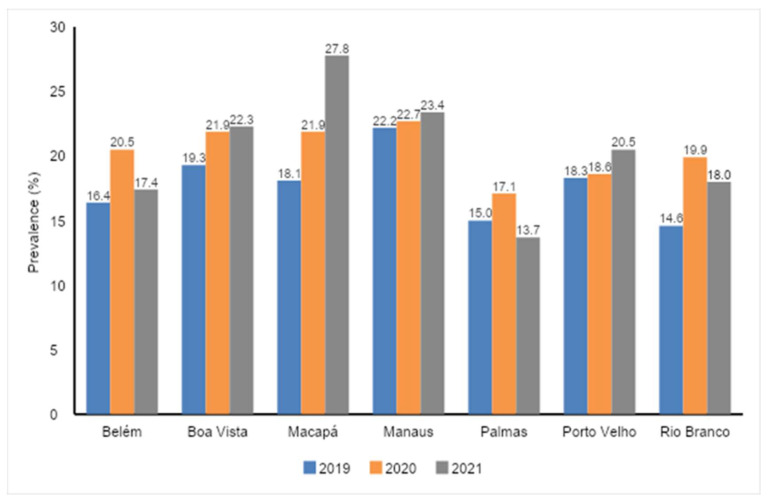
Prevalence of subgroup ≥5 consumption of ultra-processed foods on the previous day in the capitals of the Brazilian Amazon region in the years 2019, 2020, and 2021.

In relation to the prevalence of consumption of ≥5 subgroups of ultra-processed foods, studies were carried out in the capitals of the northern region of Brazil (Supplementary Table S3). It was observed that there was an increase in prevalence in all cities between 2020 and 2019, as well as in 2021 and 2019. The exception was the Palmas capital, which showed a lower prevalence between 2021 and 2019 (Figure 3).

The capital of Boa Vista exhibited a statistically significant increase of 28.5% (*p* = 0.014) in the percentage difference in subgroup consumption of ≥5 ultra-processed foods between 2019 and 2020 (Table 3). However, in 2019 and 2021, Boa Vista and Macapá showed an increase in the percentage difference of 30.4% (*p* = 0.014) and 53.7% (*p* = 0.014), respectively.

## 4. Discussion

The results of this study point to the increased frequency of consumption of ultra-processed food subgroups in the capital regions of the Brazilian Amazon between the years 2019 and 2021. With Boa Vista and Macapá as highlights, a significant increase in the consumption of packet snacks or salty crackers, cookies, and meat products was observed. These changes in eating habits during and after social distancing could have an impact on the development and worsening of chronic diseases, resulting in an increased burden on the healthcare system.

It has been observed that the consumption of ultra-processed foods is closely associated with the emergence of chronic non-communicable diseases (CNCDs) and other health problems [25,26,27,28], such as cardiometabolic diseases, common mental disorders, and all-cause mortality [16]. In this study, there was a significant increase in the intake of ultra-processed foods at the expense of fresh foods, which has substantial implications for the development of health policies and strategies, especially in critical scenarios such as the current COVID-19 pandemic.

Andrade et al. [29] found in their study an increase in the consumption of unhealthy markers, indicating changes in the eating habits of Brazilians, and these changes are related to the increase in the consumption of ultra-processed products (soft drinks, sweet biscuits, stuffed biscuits or packaged cupcakes, sausages, margarine and industrialized sauces, and ready-to-eat foods). In the present study, the cities of Boa Vista and Macapá stood out for their high consumption of ≥5 subgroups of ultra-processed foods, exceeding the average for Brazilian capitals in 2020 [30]. This increase in the frequency of consumption of these food subgroups in Brazilian capitals went from 17.8% in 2019 to 18.5% in 2020 [22].

Sarkar et al. [31] and Ferreira Rodrigues et al. [32] highlighted that the imposition of strict government measures had a significant impact on eating habits, affecting culinary preferences, purchasing, and food preparation behaviors among families.

In Italy, social distancing measures resulted in restrictions on daily access to supermarkets [3], reducing the consumption of fresh foods. In Brazil, there has been an increase in the consumption of ultra-processed foods [29,30], alcohol, and tobacco [31], associated with a rise in the incidence of depression and anxiety [32,33]. Other factors, such as regional social inequalities, may have contributed to this change. During this period, food insecurity worsened, especially in the North [34] and Northeast, where the fragility of access to essential services increased the risk of both food and nutritional insecurity and the spread of COVID-19 [35].

In this context, Ribeiro et al. [34] highlight that the frequency of consumption of ultra-processed foods during the pandemic may have been strongly influenced by factors such as unemployment, the reduction in resources allocated to social programs such as Bolsa Família, and the increase in the prices of essential foods. Additionally, these foods tend to be more affordable [22], have improved palatability, and have a longer shelf life, leading to greater population adherence to ultra-processed foods during health crises [21,36].

These findings highlight a growing trend in the preference for ultra-processed foods, both nationally [37] and globally [38]. In economically less developed regions, such as the Northeast and the North, there is a concerning tendency towards increased consumption of these foods at the expense of healthy eating habits [37]. Socioeconomic conditions, such as lower levels of education, raise concerns about the quality of dietary choices. However, this comprehensive study conducted in the years 2019, 2020, and 2021 revealed an increase in education levels in these regions, suggesting a possible disconnect between education level and healthy food consumption.

In Brazil, there is an evident divergence in recent studies regarding food consumption habits during the pandemic period, which raises pertinent questions about the quality of the population’s diet in this scenario [39]. While some other studies demonstrate an increase in the habitual frequency of fresh food intake [38], greater consumption of vegetables, fruits, and legumes is a marker of healthy eating [37]. On the other hand, other studies demonstrated an increase in the frequency of consumption of preprepared meals, savory snacks, chocolate products, and bakery items [40,41], as demonstrated in the present study.

However, it is essential to address certain limitations present in these studies. Among them, the cross-sectional design of the data collected for the years 2019, 2020, and 2021 stands out, hindering the establishment of causal inferences that could elucidate the discrepancies found in the literature. Moreover, it is noteworthy that the study was conducted via landline telephone during the specified years, potentially introducing bias, as owning a landline phone at home is often associated with socioeconomic factors.

Nonetheless, it is evident that the present study presents a certain pioneering spirit, as the existence of studies that relate to the consumption of ultra-processed foods in the North region, specifically during and after social distancing in the COVID-19 pandemic, is scarce.

In addition, transportation limitations in the northern region of Brazil present significant challenges for the distribution of fresh food, which has led to an increase in the consumption of ultra-processed food products and further aggravated socioeconomic and health challenges. The capital cities of Macapá and Boa Vista serve as illustrative examples of the logistical difficulties inherent in the region. Macapá depends on air and river transportation, with no direct road connection to the rest of the country. The city of Boa Vista is mainly accessible via the BR-174 highway and the Atlas Brazil Cantanhede International Airport, with no direct paved federal road connection to other capitals. This geographical configuration prevents the arrival of fresh food, which can contribute to a dependence on ultra-processed foods.

The North and Northeast regions of Brazil have the lowest healthy life expectancy indicators. In addition, there is a notable decline in healthy life expectancy among residents of the poorest areas, particularly among the elderly.

These findings are essential to supporting the planning and surveillance of public health policies aimed at this scenario. Since specific conditions in the region can influence food choices, this can result in health problems in the future. Although it was not the primary objective of this study to relate the consumption of UPF with diseases, it was possible to present a portrait of consumption during this pandemic period and enable discussion among society that it is necessary to think not only about food but also about access to foods of nutritional quality. 

Although some countries have already implemented taxes on ultra-processed drinks, such as sugary drinks, a few have adopted taxes on snacks or other types of UPF. Another important point that contributes to reducing consumption of these products is effective package labeling with impactful warning measures. In addition, some school feeding policies have been shown to be effective in reducing UPF consumption, collaborating significantly with public health policies [42,43]. However, there is a need for more studies that specifically evaluate the effects of increased consumption of ultra-processed foods in this region.

## 5. Conclusions

The study showed an increase in the frequency of consumption of ultra-processed foods in the Brazilian Amazon region, during and after social distancing. Furthermore, it was possible to observe the relationship between events involving health and eating habits during public health crises.

So, these findings are essential for the development of future effective public policies and strategies during a health crisis situation and can encourage future research involving the improvement of the population’s dietary pattern.

## Figures and Tables

**Table 1:** Prevalence of consumption of ultra-processed food subgroups on the previous day in the capitals of the Brazilian Amazon region in the years 2019, 2020, and 2021.

Food Group	2019	2020	2021
	% (IC95%) *	% (IC95%) *	% (IC95%) *
Soft drinks	29.3 (27.6; 30.9)	26.1 (24.0; 28.2)	28.1 (25.9; 30.4)
Fruit juice in a carton	14.5 (13.1; 15.9)	12.9 (11.4; 14.5)	13.1 (11.5; 14.7)
Soft drink powder	10.2 (9.1; 11.4)	11.1 (9.6; 12.8)	9.7 (8.3; 11.2)
Chocolate drink	11.5 (10.3; 12.7)	11.4 (9.8; 13.1)	10.3 (8.9; 11.9)
Flavored yogurt	15.9 (14.6; 17.2)	17.4 (15.7; 19.2)	15.3 (13.7; 17.0)
Packet snacks or salty crackers	24.4 (22.9; 25.9)	26.0 (24.1; 28.0)	28.2 (26.1; 30.4)
Cookie or packet cupcake	19.6 (18.3; 21.1)	22.5 (20.6; 24.7)	22.9 (20.8; 25.1)
Sweets	19.34 (17.9; 20.7)	18.7 (17.0; 20.5)	20.2 (18.5; 22.1)
Meat products	23.1 (21.6; 24.7)	26.9 (24.7; 29.1)	26.4 (24.3; 28.5)
Breads	39.3 (37.6; 41.1)	41.0 (38.8; 43.3)	40.5 (38.2; 42.8)
Mayonnaise or ketchup	17.6 (16.3; 19.0)	17.8 (16.0; 19.7)	19.2 (17.4; 21.1)
Vegetable cream	46.2 (44.5; 48.0)	48.2 (46.0; 50.5)	47.9 (45.6; 50.3)
Ready-to-eat products	7.1 (6.1; 8.2)	7.1 (5.9; 8.6)	7.7 (6.5; 9.1)

* Values calculated with sample weights.

**Table 2:** Percentage difference in the consumption of subgroups of ultra-processed foods on the previous day in the capitals of the Brazilian Amazon region in the years 2019, 2020, and 2021.

Food Group	Year	Percentage Difference (IC95%) *	*p*-Value
Soft drinks	2020–2019	−10.8 (−19.6; −2.1)	0.014
	2021–2019	−3.8 (−13.2; 5.5)	0.422
	2021–2020	7.0 (−3.4; 17.4)	0.187
Fruit juice in a carton	2020–2019	−10.9 (−24.8; 2.8)	0.120
	2021–2019	−9.6 (−23.7; 4.4)	0.178
	2021–2020	1.3 (−14.0; 16.7)	0.868
Soft drink powder	2020–2019	8.8 (−10.9; 28.6)	0.380
	2021–2019	−5.0 (−22.8; 12.7)	0.577
	2021–2020	−13.9 (−35.0; 7.2)	0.197
Chocolate drink	2020–2019	−7.3 (−18.5; 17.0)	0.935
	2021–2019	−10.2 (−26.3; 5.9)	0.214
	2021–2020	−9.4 (−28.8; 9.8)	0.336
Flavored yogurt	2020–2019	9.7 (−4.35; 23.8)	0.176
	2021–2019	−3.6 (−16.5; 9.1)	0.573
	2021–2020	−13.4 (−28.4; 1.6)	0.081
Packet snacks or salty crackers	2020–2019	6.6 (−3.8; 17.1)	0.213
	2021–2019	15.7 (4.3; 27.1)	0.007
	2021–2020	9.1 (−2.84; 21.0)	0.135
Cookie or packet cupcake	2020–2019	14.7 (1.6; 27.8)	0.027
	2021–2019	16.2 (2.6; 29.9)	0.019
	2021–2020	1.5 (−13.5; 16.6)	0.840
Sweets	2020–2019	−3.12 (−14.5; 8.3)	0.593
	2021–2019	4.8 (−7.2; 16.9)	0.431
	2021–2020	7.9 (−5.1; 21.0)	0.235
Meat products	2020–2019	16.0 (3.8; 28.2)	0.010
	2021–2019	14.0 (2.14; 25.9)	0.021
	2021–2020	−2.0 (−15.0; 10.9)	0.758
Breads	2020–2019	4.3 (−2.96; 11.5)	0.245
	2021–2019	2.8 (−4.51; 10.27)	0.445
	2021–2020	−1.43 (−9.58; 6.71)	0.730
Mayonnaise or ketchup	2020–2019	0.8 (−12.1; 13.7)	0.899
	2021–2019	8.7 (−4.8; 22.3)	0.205
	2021–2020	7.9 (−6.8; 22.7)	0.292
Vegetable cream	2020–2019	4.3 (−1.9; 10.5)	0.177
	2021–2019	3.62 (−2.8; 10.0)	0.270
	2021–2020	−0.6 (−7.7; 6.3)	0.847
Ready-to-eat products	2020–2019	0.6 (−23.5; 24.8)	0.956
	2021–2019	8.5 (−15.5; 32.6)	0.487
	2021–2020	7.8 (−18.4; 34.2)	0.558

* Values calculated with sample weights. *p*-value ≤ 0.05.

**Table 3:** Percentage difference in subgroup consumption of ≥5 of ultra-processed foods on the previous day in the capitals of the Brazilian Amazon region in the years 2019, 2020, and 2021.

City	Year	Percentage Difference (IC95%) *	*p*
Belém	2020–2019	24.6 (−7.4; 56.6)	0.133
	2021–2019	6.0 (−22.2; 34.3)	0.676
	2021–2020	−14.9 (−40.6; 10.7)	0.256
Boa Vista	2020–2019	28.5 (5.7; 51.2)	0.014
	2021–2019	30.4 (5.8; 54.9)	0.015
	2021–2020	8.3 (−17.7; 34.3)	0.532
Macapá	2020–2019	21.3 (−9.9; 52.4)	0.181
	2021–2019	53.7 (15.3; 92.1)	0.006
	2021–2020	26.7 (0.3; 53.2)	0.047
Manaus	2020–2019	2.2 (−22.4; 26.8)	0.859
	2021–2019	5.36 (−20.2; 30.9)	0.681
	2021–2020	3.0 (−25.1; 31.4)	0.831
Palmas	2020–2019	7.5 (−22.6; 37.7)	0.624
	2021–2019	−13.2 (−40.4; 13.9)	0.338
	2021–2020	−19.3 (−45.6; 6.8)	0.148
Porto Velho	2020–2019	1.85 (−26.4; 30.1)	0.898
	2021–2019	12.2 (−17.6; 42.0)	0.432
	2021–2020	10.1 (−21.4; 41.7)	0.528
Rio Branco	2020–2019	35.9 (−2.1; 74.0)	0.064
	2021–2019	23.2 (−10.4; 56.9)	0.177
	2021–2020	−9.3 (−36.0; 17.2)	0.490

* *p*-value ≤ 0.05.

## Data Availability

The data used in this study can be found on the Ministry of Health website (https://svs.aids.gov.br/daent/acesso-a-informacao/inqueritos-de-saude/vigitel/) (accessed on 30 September 2022).

## Supplementary Materials

The following supporting information can be downloaded at: https://www.mdpi.com/article/10.3390/nu16132117/s1, Table S1: Subgroups of ultra-processed foods used in the Vigitel questionnaire; Table S2: Sociodemographic characteristics of research participants in the capitals of the Brazilian Amazon region in the years 2019, 2020 and 2021; Table S3: Prevalence of consumption ≥5 subgroups of ultra-processed foods, the previous day, in the capitals of the Brazilian Amazon region in the years 2019, 2020 and 2021.
