# Supplementary material for: Consumption of Ultra-Processed Foods in the Brazilian Amazon during COVID-19
Source: Nutrients. 2024 Jul 2;16(13):2117. doi: 10.3390/nu16132117 (PMC11243564; doi:10.3390/nu16132117)
Supplement: Supplementary file 1 [file nutrients-16-02117-s001.zip › nutrients-2997466-supplementary.pdf]

Supplementary Table S1: Subgroups of ultra-processed foods used in the Vigitel questionnaire.

Soft drinks	Soft drinks
Fruit juice in a carton	Fruit juice in a box, box or can
Soft drink powder	powdered refreshment
Chocolate drink	Chocolate drink
Flavored yogurt	Flavored yogurt
Packet snack or salty crackers	Packaged snacks (or chips) or salty crackers
Cookie or packet cupcake	Biscuit/ sweet biscuit, stuffed biscuit or packaged cupcake
Sweets	Chocolate, ice cream, gelatin, flan or other industrialized dessert
Meat products	Sausage, sausage, mortadella or ham
Breads	Bread, hot dog or hamburger bun
Mayonnaise, ketchup or shown	Mayonnaise, ketchup or mustard
Vegetable cream	Margarine
Ready-to-eat products	Instant noodles (such as ramen noodles), packaged soup, frozen lasagna or other ready-to-eat dishes purchased frozen

Supplementary Table S2: Sociodemographic characteristics of research participants in the capitals of the Brazilian Amazon region in the years 2019, 2020 and 2021.

	2019		2020		2021	
	**n**	**% (IC95%)***	**n**	**% (IC95%)***	**n**	**% (IC95%)***
Sex						
Masculine	4133	47.7 (45.9; 49.5)	2752	47.73 (45.47; 49.99)	2650	47.74 (45.38; 50.12)
Feminine	7077	52.25 (50.5; 54.0)	4278	52.26 (50.00; 54.21)	4372	52.25 (49.88; 54.61)
Age range						
18–24 years old	1181	16.75 (15.5; 18.0)	731	16.33 (14.77; 18.03)	612	16.17 (14.39; 18.13)
25–34 years old	1275	28.33 (26.5; 30.1)	972	28.26 (25.99; 30.64)	921	28.22 (25.87; 30.70)
35–44 years	1757	21.04 (19.65; 22.50)	1273	21.01 (19.26; 22.88)	1290	20.94 (19.20; 22.80)
45–54 years	1925	16.49 (15.32; 17.74)	1228	16.73 (15.23; 18.34)	1199	16.83 (15.27; 18.51)
55–64 years old	2159	9.96	1272	10.16	1204	10.27

		(9.24; 10.72)		(9.22; 11.2)		(9.30; 11.32)
65 years or older	2913	7.40 (6.94; 7.88)	1554	7.48 (6.86; 8.16)	1796	7.54 (6.96; 8.16)
Years of study						
0–8 years	2565	26.36 (24.66; 28.14)	1552	25.31 (23.23; 27.50)	1592	24.61 (22.39; 26.98)
9–11 years	4011	43.86 (42.15; 45.59)	2595	44.54 (42.30; 46.80)	2695	47.19 (44.86; 49.53)
12 years and over	4634	29.76 (28.37; 31.18)	2883	30.14 (28.35; 32.00)	2735	28.18 (26.39; 30.05)
City						
Belém	2059	28.14 (29.71; 20.59)	1007	27.89 (25.82; 30.07)	1010	27.70 (25.63; 29.88)
Boa Vista	1017	5.90 (5.35; 6.50)	1003	5.93 (5.45; 6.46)	1000	5.95 (5.43; 6.52)
Macapá	973	8.02 (7.32; 8.78)	1014	8.07 (7.43; 8.77)	1002	8.11 (7.42; 8.87)
Manaus	1953	36.77 (34.92; 38.65)	1002	36.81 (34.45; 39.24)	1002	36.81 (34.29; 39.40)
Palmas	1470	5.34 (4.94; 5.78)	1000	5.42 (4.94; 5.94)	1007	5.44 (4.90; 6.03)
Porto Velho	1926	8.97 (8.31; 9.68)	1002	8.98 (8.16; 9.88)	1000	9.09 (8.24; 10.02)
Rio Branco	1812	6.83 (6.29; 7.42)	1002	6.85 (6.21; 7.56)	1001	6.86 (6.21; 7.56)

* Values calculated with sample weights. Before: referring to the year 2019. During social distancing: referring to 2020. After social distancing: referring to the year 2021.

Table S3 - Prevalence of consumption ≥ 5 subgroups of ultra-processed foods, the previous day, in the capitals of the Brazilian Amazon region in the years 2019, 2020 and 2021.

**City**	**2019 % (IC95%)***	**2020 % (IC95%)***	**2021 % (IC95%)***
Belém	16.4 (14.1; 19.1)	20.5 (16.5; 25.0)	17.4 (13.9; 21.6)
Boa Vista	19.3 (15.3; 23.9)	21.9 (18.7; 25.5)	22.3 (18.7; 26.3)
Macapá	18.1 (14.6; 22.1)	21.9 (18.7; 25.5)	27.8 (24.0; 31.9)
Manaus	22.2 (19.1; 25.6)	22.7 (18.6; 27.3)	23.4 (19.1; 28.6)
Palmas	15.9 (13.1; 19.1)	17.1 (13.8; 20.9)	13.7 (10.6; 17.6)
Porto Velho	18.3 (15.2; 21.8)	18.6 (15.3; 22.9)	20.5 (16.8; 24.8)
Rio Branco	14.6 (12.1; 17.5)	19.9 (16.0; 24.5)	18.0 (14.6; 22.0)
